# Markers of atopic dermatitis, allergic rhinitis and bronchial asthma in pediatric patients: correlation with filaggrin, eosinophil major basic protein and immunoglobulin E

**DOI:** 10.1186/s12948-018-0102-y

**Published:** 2018-11-12

**Authors:** Zafar Rasheed, Khaled Zedan, Ghada Bin Saif, Ragaa H. Salama, Tarek Salem, Ahmed A. Ahmed, Alaa Abd El-Moniem, Maha Elkholy, Ahmad A. Al Robaee, Abdullateef A. Alzolibani

**Affiliations:** 1 Department of Medical Biochemistry, College of Medicine, Qassim University, P.O. Box 6655, Buraidah, 51452 Saudi Arabia; 2 Department of Pediatrics, College of Medicine, Qassim University, Buraidah, Saudi Arabia; 3 Department of Dermatology, College of Medicine, King Saud University, Riyadh, Saudi Arabia; 4 Research Center, College of Medicine, Qassim University, Buraidah, Saudi Arabia; 5 Department of Medicine, College of Medicine, Qassim University, Buraidah, Saudi Arabia; 6 Department of Dermatology, College of Medicine, Qassim University, Buraidah, Saudi Arabia

**Keywords:** Atopic disorders, Pediatric patients, Atopic dermatitis, Allergic rhinitis, Bronchial asthma, Filaggrin, Eosinophil MBP, IgE

## Abstract

**Background:**

Allergic reactions have been implicated as contributions in a number of atopic disorders, including atopic dermatitis (AD), allergic rhinitis (AR) and bronchial asthma (BA). However, the potential for filaggrin protein, eosinophil major basic protein (MBP) and immunoglobulin E (IgE) to elicit allergic response or to contribute to atopic disorders remains largely unexplored in pediatric patients. This study was undertaken to investigate the status and contribution of filaggrin protein, eosinophil MBP and total IgE in pediatric patients with AD, AR and BA.

**Methods:**

Sera from 395 pediatric patients of AD, AR or BA with varying levels of disease activity according to the disease activity index and 410 age-matched non-atopic healthy controls were evaluated for serum levels of atopic markers, including filaggrin, eosinophil MBP and IgE.

**Results:**

Serum analysis showed that filaggrin levels were remarkably high in pediatric patients with AD, followed by BA and AR, whereas its levels were low in non-atopic pediatric controls. Eosinophil MBP levels in sera of atopic patients were significantly high as compared with their respective controls, but its levels were highest in AR patients, followed by AD and BA. Total IgE in sera of AD patients was markedly high, followed by AR and BA patients, whereas its levels were low in non-atopic pediatric controls. Interestingly, not only was an increased number of subjects positive for filaggrin protein, eosinophil MBP or total IgE, but also their levels were statistically significantly higher among those atopic patients whose disease activity scores were higher as compared with atopic patients with lower disease activity scores.

**Conclusions:**

These findings strongly support a role of filaggrin protein, eosinophil MBP and IgE in the onset of allergic reactions in pediatric patients with AD, AR and BA. The data suggest that filaggrin, eosinophil MBP or IgE might be useful in evaluating the progression of AD, AR or BA and in elucidating the mechanisms involved in the pathogenesis of these pediatric disorders.

## Introduction

The atopic disorders of childhood involve mainly atopic dermatitis (AD), allergic rhinitis (AR), and bronchial asthma (BA). These atopic disorders share a common pathogenesis, being mediated by immunoglobulin E (IgE) [1]. Frequency of these atopic disorders is on the rise and now it reached up to 20% worldwide [2]. The concept of the atopic march was developed to describe the progression of atopic disorders from AD to AR in infants or from AD to BA in children [3]. It is also reported that the onset of atopic disorders can be occurred due to a personal or familial propensity to produce sensitization or IgE antibodies in response to the environmental triggers [1, 2]. Not only environmental triggers, heritability also plays an important role in the onset of these atopic disorders in 71–84% AD patients, 33–91% AR patients and 35–95% BA patients [4, 5].

Many of the key structural proteins in the outer most layer of the epidermis involved cornification, which are encoded in a locus on chromosome 1q21, which is basically an epidermal differentiation complex (EDC) [6]. The genes found within this locus, encode for filaggrin, a key member of the EDC [6–8]. The filaggrin has now been considered as a major predisposing gene for various atopic disorders, which resulted in a major paradigm in the dermatology and allergy research [8]. Many studies pointed out an association of filaggrin gene with different atopic disorders. More specifically, mutations in the filaggrin gene have been reported to have an association with various atopic/allergic disorders [5–8]. Investigators reported that there is a strong and consistent association between filaggrin mutations and development of AD [9]. Some studies also noticed an association between filaggrin mutations with atopic sensitization in AR or BA [8, 9]. Allergic disorders are characterized by an IgE-mediated reaction as a consequence of the exposure to a specific allergen. Thus, allergen-specific IgE (sIgE) production are considered to be the hallmark of allergy [10]. Moreover, studies have also pointing out a clear association between serum IgE levels with AD or BA patients [10, 11]. Reports have also shown that children with very high serum IgE levels are at risk for anaphylactic reactions and for the occurrence of more severe AD [10]. The eosinophil major basic protein (MBP), a constituent of the eosinophil secondary granule, is implicated in cytotoxicity and mediation of allergic disorders such as asthma and the level of eosinophil MBP was reported to be high in biological fluids from patients with asthma and other eosinophil-associated disorders [12].

Even through allergic reactions have been implicated in the pathogenesis of pediatric AD, AR or BA, the potential of atopic markers such as filaggrin protein and eosinophil MBP in eliciting a pathogenic response and in contributing in the pathogenesis of these atopic disorders remains largely unexplored. In view of these, the present study was hypothesized that overproduction of filaggrin protein, eosinophil MBP and total IgE in the serum of different atopic pediatric patients may be involved in the contribution of atopic/allergic disorders. To assess this hypothesis, the levels of filaggrin protein, eosinophil MBP and total IgE were determined in the sera of pediatric patients with AD, AR and BA and their levels were compared with non-allergic pediatric controls. Our results not only support a role of filaggrin protein, eosinophil MBP and total IgE in atopic disorders, but also suggest that filaggrin, eosinophil MBP and total IgE may be important in the evaluation and in the elucidation of the mechanisms of pediatric AD, AR and BA.

## Subjects and methods

### Human subjects

This is a prospective case–control study, which enrolled atopic children with BA, AR and AD who were diagnosed according to recent guidelines described by Global Initiatives for Asthma (GINA) for BA [13], Allergic Rhinitis and its Impact on Asthma (ARIA) for AR [14] and SCORAD index for AD [15]. The study was carried out in accordance with the Code of Ethics of the World Medical Association (Declaration of Helsinki as revised in Tokyo 2004) for humans and was approved by Ethical Committee of College of Medicine, QU and study protocol was approved by the National Plan for Science, Technology and Innovation of KSA (NSTIP # 11-BIO1459-09). Informed consent from parents of all studied children was taken before samples collection. All studied atopic children were consecutively recruited from Outpatient Clinics affiliated to Qassim University (pediatric and dermatology clinics) during 1 year duration. A total of 395 allergic children, 202 males (51.1%), 193 females (48.9%), with a mean age of 6.7 years (± 1.27) were enrolled in the study. Also the study included 410 age and sex matched children who were free of any atopic disease and were used as controls. Out of 395 atopic patients, 130 patients were AD (age 1.43 ± 1.36), 120 AR patients (age 4.21 ± 1.72) and 145 were BA (age 7.42 ± 1.46). These atopic patients were further divided into two groups based on their severity scores as described previously [13–15]. Specifically, BA patients were divided into mild-moderate persistent BA and severe persistent BA on the basis of clinical features described by GINA [13], whereas AR patients were also divided into mild-moderate persistent AR and severe persistent AR on the basis of visual analog scale (VAS) as described previously [14]. In the same ways, patients with AD were also divided into mild-moderate AD and severe AD on the basis of SCORAD index [15]. Venous blood from all studied patients and controls were taken and sera were stored in − 80 °C until analyzed.

### Measurement of filaggrin protein, eosinophil major basic protein and immunoglobulin E in atopic patients

Levels of filaggrin, eosinophil MBP and total IgE were measured in the serum samples of all tested atopic patients and their levels were compared with their respective non-atopic healthy controls’ sera. Serum filaggrin levels were measured by specific human filaggrin sandwich ELISA in accordance with the manufacturers’ instructions (cat. # SEJ103Hu, Cloud-Clone Corp., Hubei, PRC.). Whereas, serum eosinophil MBP levels were measured by human eosinophil MBP specific sandwich ELISAs (cat. # SEB650Hu) according to their manufacturers’ instructions (Cloud-Clone Corp., Hubei, PRC). Whereas, total IgE serum levels were measured by human IgE specific sandwich ELISA (cat. # 20783–72876, GenWay Biotech, CA, USA).

### Statistical analysis

All statistical analysis was carried out by Graph Pad Prism version 5.0 (Graph Pad Software Inc., San Diego, CA, USA). One-way ANOVA of variance followed by Tukey–Kramer multiple comparisons test, or Two-way ANOVA of variance followed by Bonferroni comparisons test. p < 0.05 was considered significant. Results are expressed as the mean ± SEM unless stated otherwise.

## Results

### Filaggrin in different atopic disorders

**Fig. 1:**
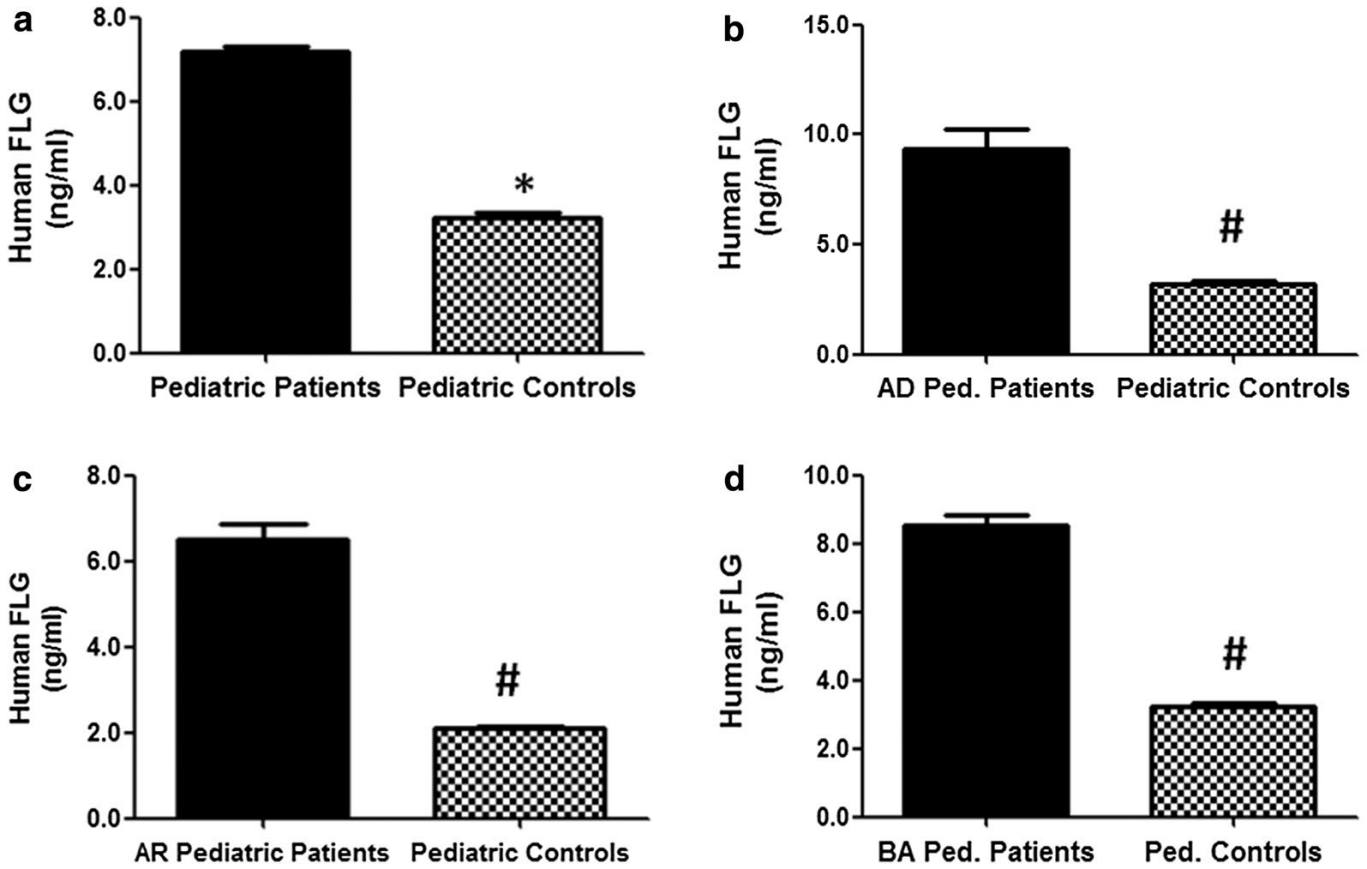
Filaggrin in pediatric patients with atopic dermatitis, allergic rhinitis and bronchial asthma. **a** Levels of human filaggrin (FLG) in the sera of all studied atopic pediatric patients (n = 395) and non-atopic pediatric controls (n = 410). *p < 0.01 versus all tested atopic patients. **b** Levels of human FLG in the patients’ sera of atopic dermatitis pediatric patients (AD Ped. Patients; n = 130) and non-atopic pediatric controls (n = 410). ^#^p < 0.001 versus AD Ped. Patients. **c** Levels of human FLG in the patients’ sera of allergic rhinitis pediatric patients (AR pediatric patients; n = 120) and non-atopic pediatric controls (n = 410). ^#^p < 0.001 versus AR pediatric patients. **d** Levels of human FLG in the patients’ sera of bronchial asthma pediatric patients (BA Ped. Patients; n = 145) and non-atopic pediatric controls (n = 410). ^#^p < 0.01 versus AR Ped. Patients

In this study, we determined the serum levels of filaggrin in pediatric patients with different atopic disorders (n = 395) and their levels were compared with their respective non-atopic healthy pediatric controls (n = 410). The data showed filaggrin levels were significantly increased in different atopic patients when compared its levels in non-atopic healthy pediatric controls (p < 0.0001). The average serum filaggrin levels (± SEM) in all studied atopic subjects and non-atopic healthy controls were 7.20 ± 0.10 and 3.21 ± 0.13 ng/ml, respectively (Fig. 1a). Specifically, the average filaggrin levels (± SEM) in the patients sera with AD (n = 130), AR (n = 120), BA (n = 145) and their respective non-atopic controls (n = 410) were 9.36 ± 0.87, 6.50 ± 0.36, 8.50 ± 0.32 and 3.21 ± 0.13 ng/ml, respectively (Fig. 1b–d). Results pointed that among all tested atopic pediatric patients, AD patients contained highest filaggrin levels followed by BA and AR patients (Fig. 1).

### Eosinophil major basic protein in different atopic disorders

**Fig. 2:**
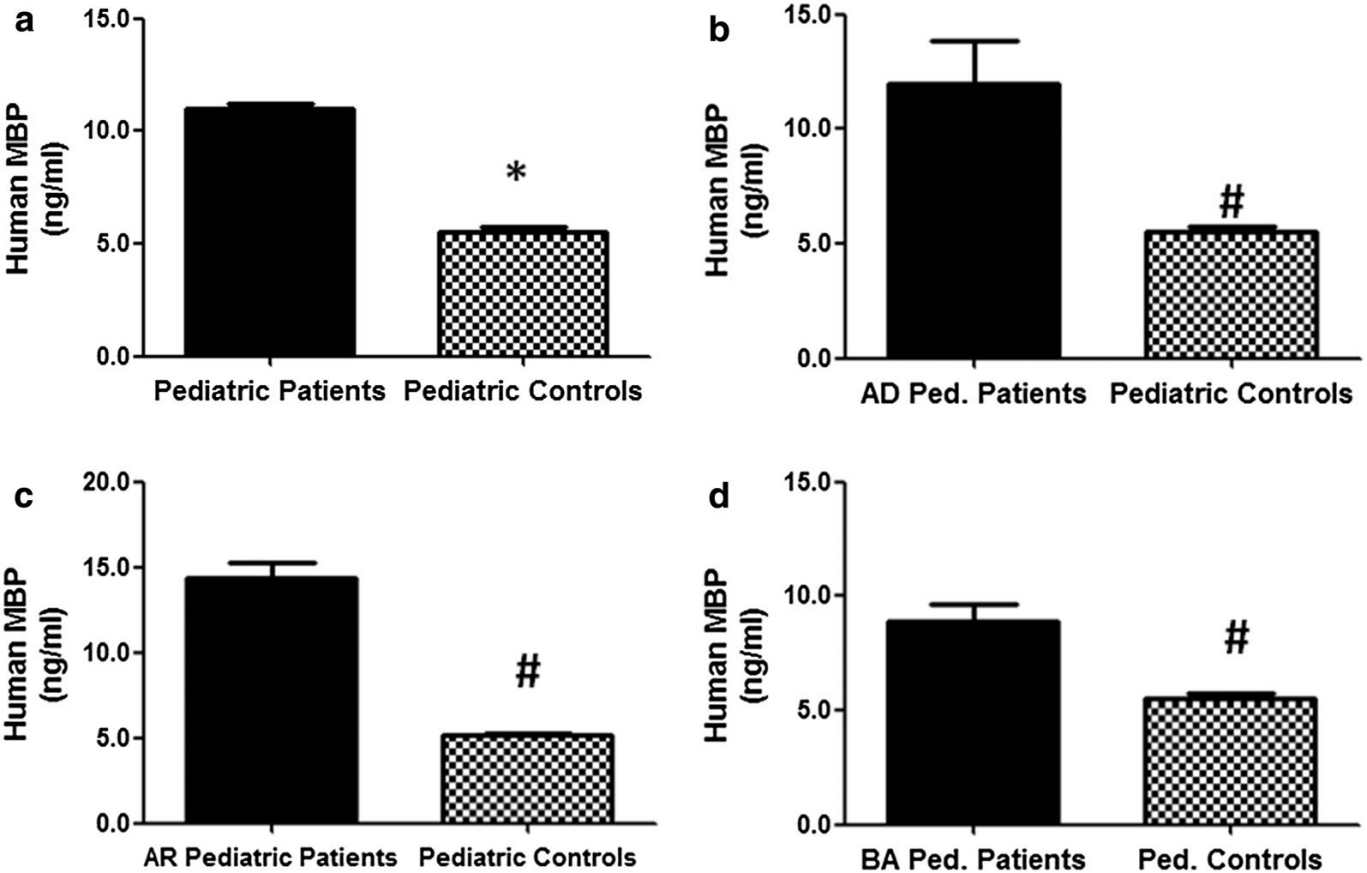
Eosinophil major basic protein in pediatric patients with atopic dermatitis, allergic rhinitis and bronchial asthma. **a** Levels of eosinophil major basic protein (MBP) in the sera of all studied atopic pediatric patients (n = 395) and non-atopic pediatric controls (n = 410). *p < 0.001 versus all tested atopic patients. **b** Levels of eosinophil MBP in the patients’ sera of atopic dermatitis pediatric patients (AD Ped. Patients; n = 130) and non-atopic pediatric controls (n = 410). ^#^p < 0.001 versus AD Ped. Patients. **c** Levels of eosinophil MBP in the patients’ sera of allergic rhinitis pediatric patients (AR pediatric patients; n = 120) and non-atopic pediatric controls (n = 410). ^#^p < 0.0001 versus AR pediatric patients. **d** Levels of eosinophil MBP in the patients’ sera of bronchial asthma pediatric patients (BA Ped. Patients; n = 145) and non-atopic pediatric controls (n = 410). ^#^p < 0.001 versus AR Ped. Patients

The serum levels of eosinophil MBP in patients with different atopic disorders (n = 395) were found to be highly significantly higher as compared with respective non-atopic healthy pediatric controls (n = 410) (p < 0.001). The average eosinophil MBP levels (± SEM) in all studied atopic subjects and non-atopic controls were 10.99 ± 0.27 and 5.54 ± 0.21 ng/ml, respectively (Fig. 2a). Importantly, the average eosinophil MBP levels (± SEM) in the patients’ sera with AD (n = 130), AR (n = 120) and BA (n = 145) were 11.97 ± 1.86, 14.39 ± 0.92 and 8.86 ± 0.77 ng/ml, respectively (Fig. 2b–d). These results not only showed the higher eosinophil MBP levels in all tested atopic patients but also pointed out that among all atopic pediatric patients, AR patients contained highest filaggrin levels followed by AD and BA patients (Fig. 2).

### Total immunoglobulin E in different atopic disorders

**Fig. 3:**
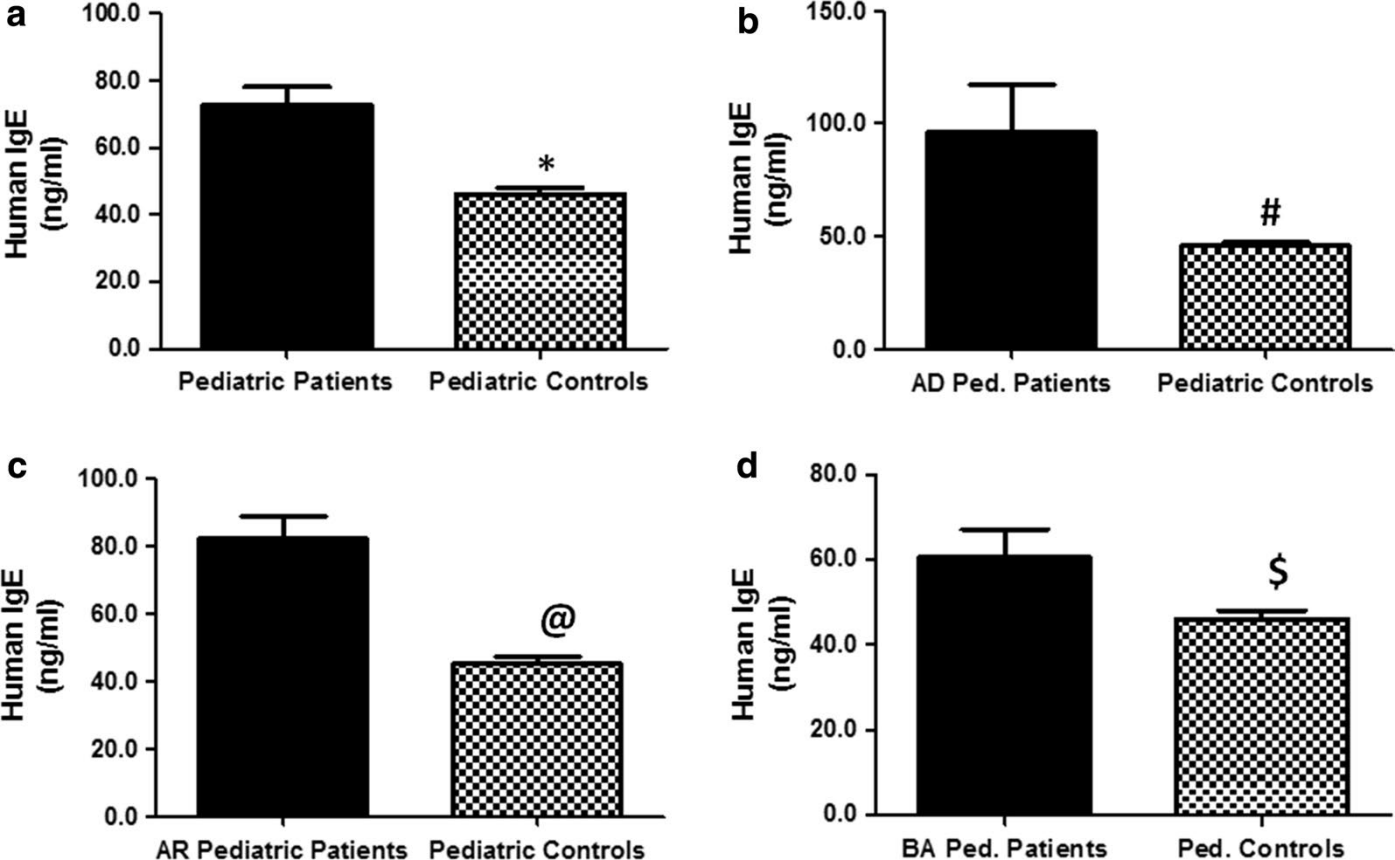
Immunoglobulin E in pediatric patients with atopic dermatitis, allergic rhinitis and bronchial asthma. **a** Levels of human immunoglobulin E (Human IgE) in the sera of all studied atopic pediatric patients (n = 395) and non-atopic pediatric controls (n = 410). *p < 0.01 versus all tested atopic patients. **b** Levels of human IgE in the patients’ sera of atopic dermatitis pediatric patients (AD Ped. Patients; n = 130) and non-atopic pediatric controls (n = 410). ^#^p < 0.001 versus AD Ped. Patients. **c** Levels of human IgE in the patients’ sera of allergic rhinitis pediatric patients (AR pediatric patients; n = 120) and non-atopic pediatric controls (n = 410). ^@^p < 0.01 versus AR pediatric patients. **d** Levels of human IgE in the patients’ sera of bronchial asthma pediatric patients (BA Ped. Patients; n = 145) and non-atopic pediatric controls (n = 410). ^$^p < 0.05 versus AR Ped. Patients

The serum levels of total IgE in patients with different atopic disorders (n = 395) were found to be significantly higher as compared with respective non-atopic healthy pediatric controls (n = 410) (p < 0.001). The average IgE levels (± SEM) in all studied atopic subjects and non-atopic controls were 72.66 ± 5.38 ng/ml and 45.95 ± 2.05 ng/ml, respectively (Fig. 3a). Specifically, the average IgE levels (± SEM) in the patients sera with AD (n = 130), AR (n = 120) and BA (n = 145) were 96.19 ± 21.3 ng/ml, 82.17 ± 6.50 ng/ml and 60.58 ± 6.56 ng/ml, respectively (Fig. 3b–d). These results not only demonstrated significant higher levels of total IgE levels in these atopic patients, but also determined that pediatric patients with AD had highest total IgE level, followed by AR and BA patients (Fig. 3b–d).

### Severity-related increase of filaggrin, eosinophil major basic protein and total immunoglobulin E in different atopic disorders

**Fig. 4:**
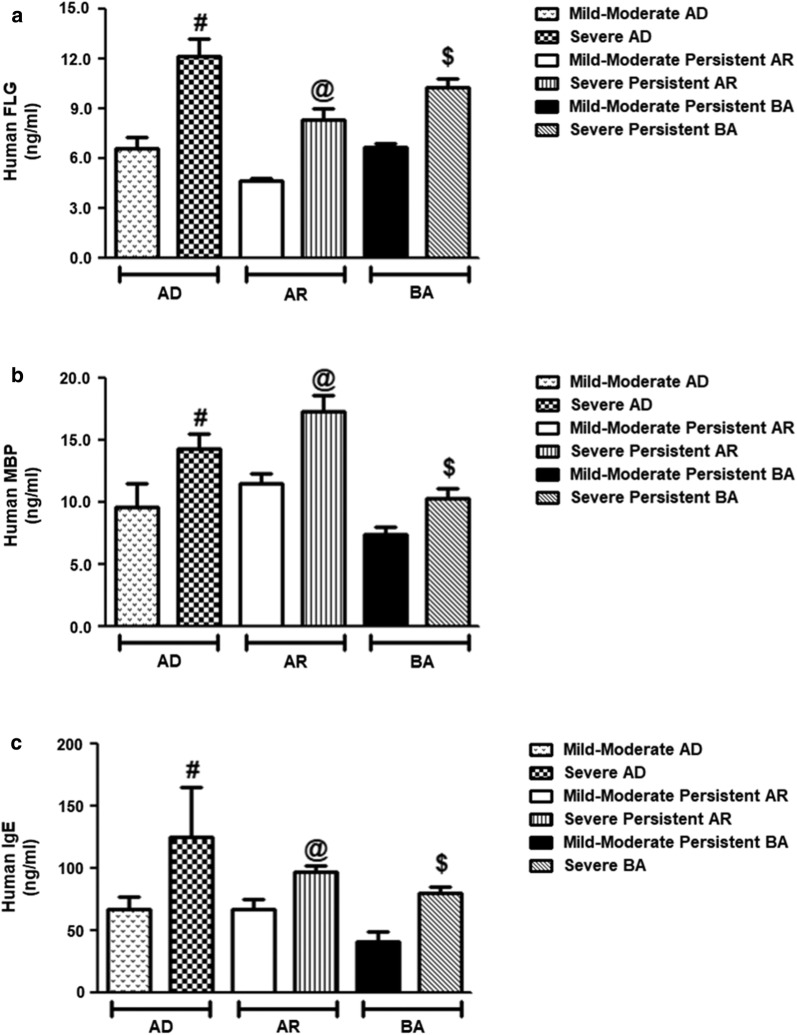
Severity-related increased of filaggrin (FLG), eosinophil major basic protein (MBP) and immunoglobulin E (IgE) in pediatric patients with atopic dermatitis (AD), allergic rhinitis (AR) and bronchial asthma (BA). **a** Levels of human FLG protein in mild-moderate AD patients’ sera (n = 97), severe AD patients’ sera (n = 33), mild-moderate persistent AR (n = 93), severe persistent AR (n = 52), mild-moderate persistent BA (n = 83), severe persistent AR (n = 37). ^#^p < 0.05 versus mild-moderate AD; ^@^p < 0.05 versus mild-moderate AR; ^$^p < 0.05 versus mild-moderate BA. **b** Levels of human MBP in mild-moderate AD patients’ sera (n = 97), severe AD patients’ sera (n = 33), mild-moderate persistent AR (n = 93), severe persistent AR (n = 52), mild-moderate persistent BA (n = 83), severe persistent AR (n = 37). ^#^p < 0.05 versus mild-moderate AD; ^@^p < 0.05 versus mild-moderate AR; ^$^p < 0.05 versus mild-moderate BA. **c** Levels of human IgE in mild-moderate AD patients’ sera (n = 97), severe AD patients’ sera (n = 33), mild-moderate persistent AR (n = 93), severe persistent AR (n = 52), mild-moderate persistent BA (n = 83), severe persistent AR (n = 37). ^#^p < 0.05 versus mild-moderate AD; ^@^p < 0.05 versus mild/moderate AR; ^$^p < 0.05 versus mild-moderate BA

To provide further support to our hypothesis and to assess the protein levels of filaggrin, eosinophil MBP and total IgE in atopic subjects, patients with AD, AR and BA were divided into different groups based on their severity scores. The average human filaggrin (±) SEM in patients’ sera with mild-moderate AD (n = 97), severe AD (n = 33), mild-moderate persistent AR (n = 83), severe persistent AR (n = 37), mild-moderate persistent BA (n = 93) and severe persistent BA (n = 52) was 6.58 ± 0.71, 12.14 ± 1.08, 4.69 ± 0.15, 8.31 ± 0.69, 6.68 ± 0.24 and 10.32 ± 0.51 ng/ml, respectively (Fig. 4a). Whereas, the average human eosinophil MBP (±) SEM in patients’ sera with mild-moderate AD (n = 97), severe AD (n = 33), mild-moderate persistent AR (n = 83), severe persistent AR (n = 37), mild-moderate persistent BA (n = 93) and severe persistent BA (n = 52) was 9.60 ± 1.96, 14.34 ± 1.21, 11.47 ± 0.81, 17.31 ± 1.31, 7.41 ± 0.62 and 10.31 ± 0.82 ng/ml, respectively (Fig. 4b). The average human total IgE (±) SEM in patients’ sera with mild-moderate AD (n = 97), severe AD (n = 33), mild-moderate persistent AR (n = 83), severe persistent AR (n = 37), mild-moderate persistent BA (n = 93) and severe persistent BA (n = 52) was 67.18 ± 10.31, 125.21 ± 40.31, 66.97 ± 8.31, 97.37 ± 5.19, 40.93 ± 7.82 and 80.23 ± 5.25 ng/ml, respectively (Fig. 4c). These results demonstrated that filaggrin protein, eosinophil MBP or total IgE were significantly increased in the serum of patients with severe atopic disorders when compared with mild-moderate patients (p < 0.05).

## Discussion

This study demonstrated the role of filaggrin protein, eosinophil major basic protein and immunoglobulin E in the pathogenesis of pediatric patients with atopic dermatitis, allergic rhinitis and bronchial asthma. The filaggrin is a key structural protein required for the normal biogenesis and physiology of the stratum corneum [16]. It is now well documented that genetic variants of filaggrin was found in up to 50% of AD patients, which enhanced our understanding on the role of filaggrin, not only in the skin barrier defect or AD pathogenesis but also in the subsequent progression of an atopic march [17]. The atopic march concept describes the progression of atopic disorders from AD to AR in infants and from AD to BA in childhood [3, 17]. The mutations in filaggrin gene have now been considered as a major risk factor for AD onset especially in pediatric patients [18]. Furthermore, not only in AD patients, filaggrin gene mutations have also been reported in patients with AR and BA [19, 20]. Importantly, it has also been shown that almost 50% of all severe cases of eczema may have at least one mutated filaggrin gene [20–23]. Truncation mutations R501X and 2284del4 are considered to be the most common mutations among the white population especially a population from the Tayside and Dumfries of Scotland [21, 22]. In addition, the barrier defect has also seen in filaggrin null which carriers also appears to lead to increased asthma susceptibility and exacerbations. Filaggrin deficiency is one of the top genome-wide genetic determinants of asthma, along with the variants found that regulate ORMDL3 expression [20–23]. In view of these, the present study was hypothesized to investigate whether mutations in filaggrin gene also effect on its protein level in the serum of BA and other allergic disorders. To the best of our knowledge, the present study for the very first time showed elevated serum levels of filaggrin protein, not only in AD and BA pediatric patients but also in patients with AR. Specifically, the data from the present study show that the filaggrin in the serum of these pediatric patients was significantly increased as compared with their non-atopic healthy pediatric controls. Not only have these, data also pointed out that filaggrin levels were markedly increased in children with AD, followed by BA and AR patients. An increased of serum levels of filaggrin in these atopic patients not only suggesting its dysfunctionality at protein level, but also indicates its potential role in the pathogenesis and/or progression of these atopic disorders.

Increased of filaggrin gene mutations has previously been detected in AD, AR and BA, but the significance of serum levels of filaggrin protein in the initiation and development explaining the mechanism of these atopic disorders remains largely unexplored. In this study, when the AD, AR or BA patients were divided into two groups (mild-moderate and severe) based on their severity scores [13–15], both groups showed higher serum levels of filaggrin protein than were observed in healthy controls, but the levels were much greater in the severe patients groups, suggesting the involvement of filaggrin protein in ongoing atopic disorders. These findings strongly support the role of filaggrin protein in the pathogenesis of AD, AR or BA and in the subsequent progression along with the atopic march. Our results not only further support the potential role of filaggrin protein in these atopic disorders, but also suggest that targeting serum levels of filaggrin may be useful in predicting the severity status of atopic disorders in pediatric patients.

Eosinophils are well known for their vital role in allergic inflammatory processes and the eosinophil major basic protein is the most abundant constituent of the eosinophil secondary granules [24]. Evidences from previous studies have shown that the eosinophil and its granular proteins might be assumed to mediate the hypersensitivity disorders [24]. In this study, the serum levels of eosinophil MBP were determined in different atopic pediatric patients and were found to be significantly high as compared with their controls. Moreover, our results also pointed out that out of all tested atopic patients, eosinophil MBP levels were found to be highest in AR patients, followed by AD and BA. In a line with our results, a previous study on tissue damage has shown that MBP has direct association with eosinophil infiltration in BA pediatric patients [24]. In addition, activated eosinophils and depositions of eosinophil granular proteins were found in AD skin biopsies [25]. Furthermore, studies have also reported higher levels of MBP in biological fluids of asthmatic patients [26]. All these studies supported our findings and clearly suggesting a direct role of eosinophil MBP in pediatric patients with atopic disorders. To further validate our central hypothesis that initiation of AD, AR or BA may be mediated by increased eosinophil’s MBP, serum eosinophil’s MBP was determined in mild-moderate atopic patients and their results were compared with severe atopic patients. Our novel results showed marked increased of eosinophil’s MBP in severe atopic patients as compared with mild-moderate atopic patients. Increased levels of eosinophil’s MBP in severe patients with AD, AR or BA, suggesting another important potential mechanism involved in the pathogenesis and/or progression of these atopic disorders.

Atopic disorders in pediatric patients can be a familial or familial propensity, which involved in the production of IgE antibodies and its associated sensitization in response to various known or unknown factors particularly environmental triggers, which linked with atopic march especially in pediatric patients [27]. The IgE sensitization and severity of atopic disorders have been well connected with each other, particularly in AD progression and BA persistence [28]. It is important to point out that it is reported that many patients with allergic disorders have elevated levels of total IgE [28–32]; however, there is no specific cutoff value that discriminates patients with allergic disorders from those without, and there is considerable overlap. Thus, total IgE by itself is rarely adequate to diagnose allergic disorders. Studies have shown that adults with IgE greater than 66 IU/ml have a 37-fold greater risk of having allergen-specific IgE antibodies to aeroallergens compared with those patients with the lowest levels of total IgE [29]. In one study, school-aged children had a mean IgE level of 51 IU/ml. Children with both AD and asthma had mean IgE levels of 985 IU/ml; those with asthma alone, 305 IU/ml; those with eczema alone, 273 IU/ml, and those with AR, 171 IU/ml [30]. Using a total serum IgE level of 100 IU/ml to discriminate allergic from non-allergic patients, the sensitivity was 78% for patients with asthma and 60% for AR, but 20% of patients were misclassified [32]. Although there is no specific cut off value of total IgE for atopic disorders but it’s abnormally is well reported, therefore it assumes to be a useful biomarker for the diagnosis of AD, AR or BA in addition with other atopic markers as defined here. In the current study, we found that higher levels of total IgE in different atopic disorders as compared with their respective non-atopic pediatric controls. Most importantly, our data also pointed out that out of all studied atopic disorders in children, total IgE levels were highest in AD patients, followed by AR and BA. The enhanced total IgE levels observed in atopic patients in this study drew our attention to evaluate its levels in different atopic subjects with varying disease severity. Our data clearly showed that the total IgE levels were significantly higher in the group of patients with high disease severity scores as compared with mild-moderate disease groups of AD, AR or BA pediatric patients. The increased of serum levels of total IgE in severe atopic patients suggest that IgE levels in the serum of these subjects are associated with increased disease activity in these atopic patients. These results are well supported by previous findings, showed that eczema patients with IgE antibodies against common environmental allergens were at higher risk for the development/progression of atopic disorders especially in pediatric patients [33, 34]. The increased levels of IgE observed in the patients with AD, AR or BA in the present study, together with significant increases of filaggrin protein and eosinophil MBP, provide strong evidences of their involvement in these disorders and clearly indicating that filaggrin protein, eosinophil MBP and total IgE are useful in evaluating of AD, AR or BA disease activity, and would therefore be helpful for predicting the progression of these atopic disorders in pediatric patients.

## Conclusions

Our data clearly show that the levels of filaggrin protein, eosinophil MBP and total IgE were increased in pediatric patients with atopic dermatitis, allergic rhinitis and bronchial asthma. These results conclude that serum levels of filaggrin, eosinophil MBP and total IgE might be useful in the diagnosis of these atopic disorders in pediatric patients. Longitudinal studies in atopic pediatric patients are necessary to invent novel agents that can antagonize or modify the effects of filaggrin, eosinophil MBP or total IgE on the atopic march, thereby we can better treat, prevent or control these atopic disorders especially in pediatric patients.
